# Gender health gap pre- and post-joint arthroplasty: identifying affected patient-reported health domains

**DOI:** 10.1186/s12939-024-02131-5

**Published:** 2024-02-27

**Authors:** Viktoria Steinbeck, Anja Yvonne Bischof, Lukas Schöner, Benedikt Langenberger, David Kuklinski, Alexander Geissler, Christoph Pross, Reinhard Busse

**Affiliations:** 1 Department of Healthcare Management, School of Economics and Management, Technical University Berlin, Strasse Des 17 Juni 135, Berlin, 10623 Germany; 2 School of Medicine, Chair of Health Economics, Policy and Management, University of St. Gallen, St. Gallen, Switzerland

**Keywords:** Gender health gap, Patient-reported outcomes, Health disparities

## Abstract

**Background:**

As patient-reported outcomes (PROs) gain prominence in hip and knee arthroplasty (HA and KA), studies indicate PRO variations between genders. Research on the specific health domains particularly impacted is lacking. Hence, we aim to quantify the gender health gap in PROs for HA/KA patients, differentiating between general health, health-related quality of life (HrQoL), physical functioning, pain, fatigue, and depression.

**Methods:**

The study included 3,693 HA patients (1,627 men, 2,066 women) and 3,110 KA patients (1,430 men, 1,680 women) receiving surgery between 2020 to 2021 in nine German hospitals, followed up until March 2022. Questionnaires used were: EQ-VAS, EQ-5D-5L, HOOS-PS, KOOS-PS, PROMIS-F-SF, PROMIS-D-SF, and a joint-specific numeric pain scale. PROs at admission, discharge, 12-months post-surgery, and the change from admission to 12-months (PRO-improvement) were compared by gender, tested for differences, and assessed using multivariate linear regressions. To enable comparability, PROs were transformed into z-scores (standard deviations from the mean).

**Results:**

Observed differences between genders were small in all health domains and differences reduced over time. Men reported significantly better health versus women pre-HA (KA), with a difference of 0.252 (0.224) standard deviations from the mean for pain, 0.353 (0.243) for fatigue (PROMIS-F-SF), 0.327 (0.310) for depression (PROMIS-D-SF), 0.336 (0.273) for functionality (H/KOOS-PS), 0.177 (0.186) for general health (EQ-VAS) and 0.266 (0.196) for HrQoL (EQ-5D-5L). At discharge, the gender health gap reduced and even disappeared for some health dimensions since women improved in health to a greater extent than men. No gender health gap was observed in most PRO-improvements and at month 12.

**Conclusions:**

Men experiencing slightly better health than women in all health dimensions before surgery while experiencing similar health benefits 12-months post-surgery, might be an indicator of men receiving surgery inappropriately early, women unnecessarily late or both. As studies often investigate the PRO-improvement, they miss pre-surgery gender differences, which could be an important target for improvement initiatives in patient-centric care. Moreover, future research on cutoffs for meaningful between-group PRO differences per measurement time would aid the interpretation of gender health disparities.

**Trial registration:**

German Register for Clinical Trials, DRKS00019916, 26 November 2019.

**Supplementary Information:**

The online version contains supplementary material available at 10.1186/s12939-024-02131-5.

## Background

In many medical fields, health differences can be observed based on sex and/or gender, the so-called sex/gender health gap. Prominent examples are differences in life expectancy [1] and chronic pain prevalence [2]. Moreover, research in different medical domains has revealed biases in the way patients are diagnosed, treated, and cared for due to their sex and gender, e.g., medical guidelines based on predominantly male symptoms that lead to under- and late diagnoses of females [3, 4] and under or non-representation of women in clinical trials [5]. Gender and sex biases sometimes get replicated, for instance, by feeding biased datasets or guidelines into decision tools, leading to outdated or misleading diagnoses and recommendations [6]. While women appear to be disproportionally affected by revealed sex and gender biases in medicine, also men suffer from these biases, e.g., in the under-diagnosis of mental illnesses [7] or osteoporosis [8]. Non-binary individuals are rarely considered in analyses at all [9].

One option to measure gender health disparities aka a potential gender health gap is the utilization of Patient-Reported Outcome Measures (PROMs). PROMs are validated questionnaires that assess health from the patient’s perspective [10]. The results of the PROMs can be called Patient-Reported Outcomes (PROs). However, outcome measurement in health systems currently often focuses on clinician and administratively reported data, hence PROs are not widely available and consequently not always used when investigating the gender health gap. However, over the last decade PROs have been used more and more in some medical fields including orthopedics [11].

Hip and knee arthroplasties (HA and KA) are the most common orthopedic surgeries worldwide with OECD averages in 2019 being 174 per 100,000 population for HA, and 137 per 100,000 for KA [12]. Germany ranked on top of the list that year for HA, with 315 surgeries per 100,000 population, and fourth highest for KA with 227 per 100,000. One of the main reasons for HA and KA is the treatment of end-stage osteoarthritis [13]. As the prevalence of osteoarthritis is higher in women [14], they receive HA and KA more frequently [14].

In orthopedic studies, PROMs are often either not reported separately by gender or are presented as secondary findings [15]. Consequently, these studies fail to adequately account for confounding factors when analyzing gender-related health differences [6]. Moreover, evidence concerning which health dimensions are affected by gender-related disparities and whether these differences can be considered meaningful is rare in recently published literature [16–20]. In addition, analyses concerning the gender health gap in HA and KA in the context of the German healthcare system, to our knowledge, do not yet exist. Since systematic gender differences in health can play a role in the dynamic of under- and oversupply of healthcare services (i.e. low-value care), an evaluation per country can give specific insights into how to adjust care in the national setting.

Hence, this paper aims to address this research gap by evaluating whether a gender health gap exists pre-surgery, at discharge, 12-months post-HA and KA, and over time from admission to 12-months post-HA and KA (PRO-improvement). Data from a large German multicenter randomized-controlled trial was used to investigate the research question for multiple generic and disease-specific PROMs covering general health, health-related quality of life (HrQoL), depression, fatigue, pain and physical functioning.

## Methods

### Dataset

In this retrospective cohort study, we make secondary use of a dataset originating from the PROMoting Quality study [21], which covered primary HA and KA patients across nine hospitals in Germany in a randomized controlled trial. Patients underwent surgery between 2020 and 2021 and were followed up until March 2022. Patients received generic and treatment-specific PROMs, at admission to the hospital, at discharge, and 12-months post-surgery. The intervention of the randomized controlled trial started at month 1 post-surgery. Further information on the PROMoting Quality trial can be found elsewhere [21, 22]. We control for the PROMoting Quality intervention when looking at the PRO-improvement and 12-month PRO values together with other control variables to distill the gender health gap.

**Fig. 1:**
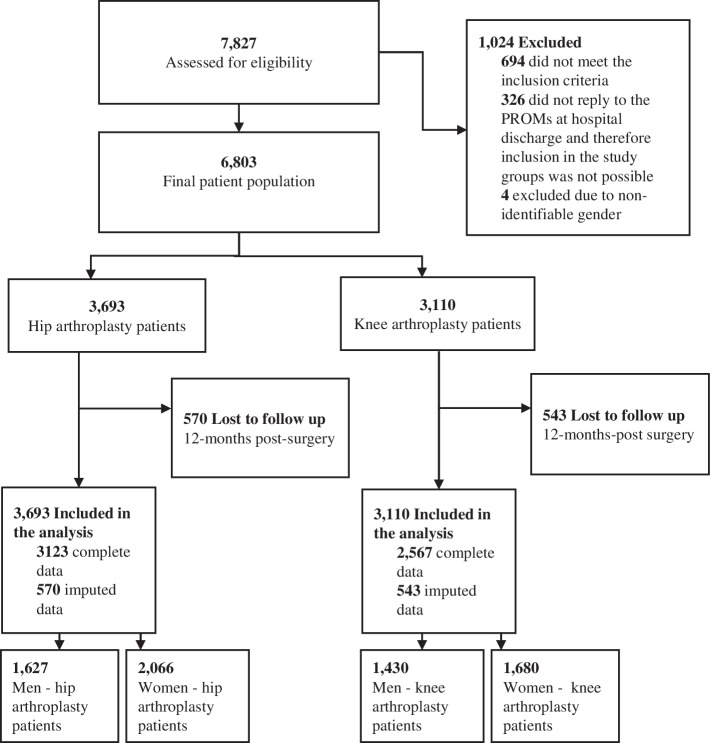
Participant flow

The selection criteria for this study are detailed in Fig. 1 and were based on the inclusion criteria of the trial [21] and the availability of the gender variable. Due to the non-representation of diverse individuals, this study focuses on the gender differences between individuals identifying as men and women only. The gender variable was retrieved from the information patients had to self-report. Other missing data besides the gender variable was imputed using the MissForest Package in R Version 4.1.3.

### Patient-reported outcome measures

General health was measured via the generic PROMs European Quality of Life Visual Analogue Scale (EQ-VAS) and HrQoL was measured using the 5-level EQ-5D version (EQ-5D-5L) [23]. The EQ-VAS ranges from 0–100 and the EQ-5D-5L ranges from -0.661 to 1, with higher scores indicating better health. We utilized the treatment specific PROMs Hip disability/Knee injury and Osteoarthritis Outcome Score Physical Function Shortform (HOOS-PS / KOOS-PS) respectively for HA and KA patients. Both cumulate in a score on a scale from 0–100 from “no difficulty” to “extreme difficulty”, meaning that lower scores indicate better physical health. The Patient Reported Outcomes Measurement Information System Fatigue Shortform (PROMIS-F-SF) measuring fatigue and the PROMIS Depression Shortform (PROMIS-D-SF) measuring depression symptoms both cover four questions and are summarized to a score from 33.7–75.8 and 41–79.4 respectively, from no fatigue/ depression to extreme fatigue/ depression [24]. Pain in the operated joint was measured on a numeric rating scale from 1 to 10, from lowest to highest possible pain. All patients were asked about the pain in the right hip joint, the left hip joint, the right knee joint and the left knee joint. We utilized the answer for the operated joint instead of a cumulative score of the answers for all pain questions. This was done because utilizing the pain scores for all four joints would have downplayed the severity of pain experienced, especially given that the pain in the operated joint is often most relevant for the decision to perform surgery and also where the most pronounced impact after surgery is expected. In case the surgery was performed on both sides (e.g. the right and left hip joint) we used the average pain of both sides (this applied to 123 HA patients and 104 KA patients). All PROMs were used in the validated German versions [24–29].

### Statistical analyses

Statistical gender differences – raw data: First, summary statistics of patient and treatment characteristics are displayed and compared between genders, using t-test and Chi-square test for continuous and categorical variables respectively. Similarly, the PROs at admission, at discharge and 12-months post-surgery were analyzed per gender. Due to non-normal distribution of PROs, the Mann–Whitney U test was utilized to test for independence between genders. This enabled the analysis of statistically significant differences in PROs between genders.

Meaningful gender differences – raw data: In addition, we analyzed whether the differences in PRO-improvement can be considered meaningful based on previously published minimally clinical important difference (MCID) thresholds. Where possible we utilized the MCIDs calculated based on the same German HA (KA) dataset which were 0.17 (0.20) for EQ-5D-5L, 7.81 (5.86) for EQ-VAS, -10.01 (-5.06) for HOOS-PS (KOOS-PS) [30] and -0.9 (-0.7) for pain [31]. For the PROMIS measures we used a 2-point cutoff [32].

Statistical gender differences – adjusted for confounders: For the following main analyses, PROs per measurement time and for the PRO-improvement were transformed into z-scores to make them comparable on one scale. Z-scores express how many standard deviations (SD) an individual’s outcome is above or below the average of the population and is calculated as:

1 $$z\frac{x-\mu }{\sigma }$$

where $x$ is the observed score per measurement time (or improvement score) of an individuum, $\upmu$ is the population’s mean and $\sigma$ its SD. In addition, some PROs were adjusted in their directionality so that for all scores a positive coefficient indicates better health. As main analyses, linear multiple regression models were run per PRO controlling for a different set of variables depending on the timepoint to distill the contribution of gender towards the different health dimensions.

For the admission PROs as dependent variable, the control variables were age in years, body mass index (BMI) group (“underweight”, “normal”, “overweight”, “obese”), education (“no school degree”, “primary school degree”,”high/middle school degree”, “university degree”), living situation (“I live with a partner/family/friends”, “I live alone”, “I live in a care facility”, “Other”) and having had one of the following comorbidities (yes/no answer options per comorbidity: heart-, circulation-, blood-, or lung-related diseases, stroke, diabetes, neurological diseases, cancer, depression, back pain and arthritis). For the discharge score as dependent variable, the previously listed information, the mobilization after surgery, the experience of the main surgeon in numbers of surgeries and the admission PRO were controlled for. For the 12-months score, in addition to the previously mentioned variables, the PRO-monitoring group [21] and the rehabilitation were controlled for. For the PRO-improvement (change from admission to month 12), we controlled for the same variables as in the 12-month regressions.

Meaningful gender differences – adjusted for confounders: As suggested by Norman et al. [33] and Bloom et al. [34], above 0.5 standard deviations can be considered as MCID across different PROMs. Hence, this threshold was used to interpret whether the differences in PRO-improvement can be considered meaningful.

All analyses were conducted in R, version 4.1.3. Due to different procedures and recovery times, all analyses were run separately for HA and KA patients.

## Results

### Patient characteristics

The study population consists of 3,693 HA patients (1,672 men and 2,066 women) and 3,110 KA patients (1,430 men and 1,680 women) as shown in Fig. 1. The descriptive statistics are presented in Table 1. HA patients identifying as women in this study are, on average 66 years old, one year older compared to patients identifying as men, show significant differences in their BMI, e.g., more frequently have a “normal” BMI when being admitted for surgery (38% versus 23%) and less often have a university degree (24% versus 35%). In addition, more HA patients who identify as women live alone (30% versus 14%), and were in employment prior to surgery (36% versus 30%). KA patients were on average 66 years old, not showing significant differences between genders. 50% of KA patients who identify as women were obese and 42% of KA patients who identify as men. Most KA patients have a high school degree, while significant gender differences were observable (87% of men and 73% of women). The PROs at admission generally show worse health levels in women e.g. with the HOOS-PS being 50.43 in women and 44.39 in men. Significant differences were observable between genders in most of the recorded commodities but not in the PRO-monitoring group (Supplementary material).

**Table 1:** Descriptive statistics of the study population (*n* = 6,803)

	Hip arthroplasty patients		*p*^b^	Knee arthroplasty patients		*p*^b^
	men (*N* = 1,627)	women (*N* = 2,066)		men (*N* = 1,430)	women (*N* = 1,680)	
Age						
mean (SD)	65.18 (10.54)	66.24 (10.59)	0.003	66.06 (9.19)	65.99 (9.26)	0.843
BMI^a^						
Underweight	0 (0%)	20 (0.97%)	< 0.001	2 (0.14%)	4 (0.24%)	< 0.001
Normal	379 (23.29%)	786 (38.04%)		191 (13.36%)	286 (17.02%)	
Overweight	742 (45.61%)	674 (32.62%)		637 (44.55%)	547 (32.56%)	
Obese	506 (31.10%)	586 (28.36%)		600 (41.96%)	843 (50.18%)	
Education						
No school degree	6 (0.37%)	9 (0.44%)	< 0.001	6 (0.42%)	12 (0.71%)	< 0.001
Primary school degree	222 (13.64%)	270 (13.07%)		266 (18.60%)	268 (15.95%)	
High/middle school degree	831 (51.08%)	1,287 (62.29%)		766 (53.57%)	1,093 (65.06%)	
University degree	568 (34.91%)	500 (24.20%)		392 (27.41%)	307 (18.27%)	
Living situation						
Alone	220 (13.52%)	624 (30.20%)	< 0.001	172 (12.03%)	427 (25.42%)	< 0.001
Care facility	3 (0.18%)	8 (0.39%)		7 (0.49%)	12 (0.71%)	
With a partner/family/friends	1,392 (85.56%)	1,423 (68.88%)		1,247 (87.20%)	1,225 (72.92%)	
Other	12 (0.74%)	11 (0.53%)		4 (0.28%)	16 (0.95%)	
Job						
Working	579 (35.59%)	621 (30.06%)	< 0.001	475 (33.22%)	447 (26.61%)	< 0.001
Voluntarily not working including retirement	866 (53.23%)	1230 (59.54%)		772 (53.99%)	1,018 (60.60%)	
Looking for work	18 (1.11%)	17 (0.82%)		15 (1.05%)	19 (1.13%)	
Unable to work	164 (10.08%)	198 (9.58%)		168 (11.75%)	196 (11.67%)	
Admission PRO^c^ mean (SD)						
EQ-5D-5L	0.644 (0.239)	0.562 (0.269)	< 0.001	0.665 (0.230)	0.589 (0.262)	< 0.001
EQ-VAS	59.14 (19.90)	54.96 (19.67)	< 0.001	61.09 (19.29)	55.91 (18.92)	< 0.001
HOOS-PS/ KOOS-PS	44.29 (15.50)	50.43 (16.00)	< 0.001	40.62 (12.67)	45.13 (12.35)	< 0.001
PROMIS-D-SF	47.96 (7.78)	51.20 (8.40)	< 0.001	47.42 (7.69)	51.04 (8.39)	< 0.001
PROMIS-F-SF	46.92 (9.20)	50.97 (10.00)	< 0.001	46.28 (9.51)	49.98 (9.80)	< 0.001
Pain in joint	6.09 (2.19)	6.72 (2.05)	< 0.001	6.49 (2.04)	7.04 (1.93)	< 0.001

^a^*BMI* body mass index

^b^for continuous variables a two-sided t-test was performed, for and categorical variables a Chi-square test was performed and for the PRO-scores at admission, a Mann–Whitney U test

^c^*PRO* Patient-reported Outcome. The score ranges are: -0.661 to 1.0 for the EQ-5D-5L, 0–100 for the EQ-VAS, HOOS-PS and KOOS-PS, 33.7–75.8 for the PROMIS-F-SF, 41–79.4 for the PROMIS-D-SF and 0–10 for pain. The EQ-5D-5L is reported with three digits after the decimal point due to the small score range

### Raw gender health gap

Table S2 and 3 in the supplementary file show the descriptive statistics of the PROs per observation time and as improvement, the mean difference between genders in points as well as the results of the significance tests. The tables also indicate whether the raw gender difference in PRO-improvement constitute MCIDs.

The results reflect significant differences between genders in almost all PROs for both joint replacement types at each timepoint, with men experiencing significantly better health across PROs. Solely in the pain dimension 12-months post HA, no significant gender difference is observable. Mean PRO differences between HA patients identifying as men and women show the widest disparities in PROs pre-surgery (HrQoL -0.081, general health -4.18, physical functioning 6.13, depression 3.25, fatigue 4.05, pain 0.63), followed by those at discharge (HrQoL -0.025, general health -2.18, physical functioning 5.88, depression 2.50, fatigue 3.15, pain 0.27) and the lowest at month 12 (HrQoL -0.021, general health -1.27, physical functioning 1.89, depression 1.70, fatigue 1.26, pain 0.04). For KA patients, the widest differences are also observable at admission (HrQoL -0.076, general health -5.19, physical functioning 4.51, depression 3.61, fatigue 3.61, pain 0.55), while the disparities are lower at discharge (HrQoL -0.030, general health -2.80, physical functioning 2.21, depression 3.17, fatigue 3.39, pain 0.46) and month 12 (HrQoL -0.030, general health -2.83, physical functioning 2.96, depression 2.51, fatigue 2.00, pain 0.18) than at admission. However, disparities do not reduce in all PROs from discharge to month 12 among KA patients. Comparing the two joint replacement types, the gender health gap is larger among HA patients at admission, but larger at discharge among KA patients in all PROs besides the physical functioning score. At month 12 the gap is larger among KA patients compared to HA patients in all PROs.

The PRO improvement from admission to month 12 shows men experience significantly less improvement in all PROs in HA and KA, with point differences of e.g. 0.061 (0.046) in HrQoL for HA (KA), -4.25 (-1.55) in physical functioning and -2.79 (-1.61) in fatigue. Based on previously mentioned MCID thresholds, only the difference in PRO-improvement in fatigue among HA patients can be considered clinically meaningful whereas the other differences in improvement are not.

### Main analyses: gender health gap after adjusting for confounders

**Fig. 2:**
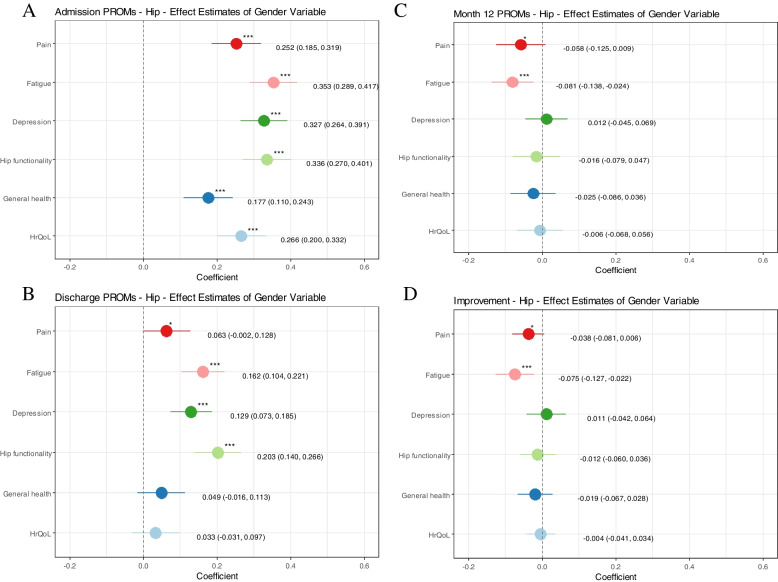
Hip arthroplasty: Explanatory power of the patients’ gender on the z-score standardized Patient-reported outcomes (PROs) per measurement time. All graphs: the outcome variables (PROs) are transformed into z-scores and adjusted in their directionality so that all scores indicate better health in men if the coefficient is positive; HrQoL = Health-related quality of life. **Graph A** - admission: results of multivariate linear regression models controlling for age, education, BMI, living situation and nine comorbidities; **Graph B** - discharge: results of multivariate linear regression models controlling for age, Body Mass Index (BMI), the respective PRO at admission, the hospital, the mobilization after surgery, the experience of the main surgeon and nine comorbidities; **Graph C** - month 12: results of multivariate linear regression models controlling for age, BMI, the respective PRO at admission, the mobilization after surgery and the experience of the main surgeon, the monitoring group, the rehabilitation form and nine comorbidities **Graph D** - PRO-improvement: results of multivariate linear regression models controlling for age, BMI, the respective PRO at admission, the mobilization after surgery and the experience of the main surgeon, the monitoring group, the rehabilitation form and nine comorbidities

**Fig. 3:**
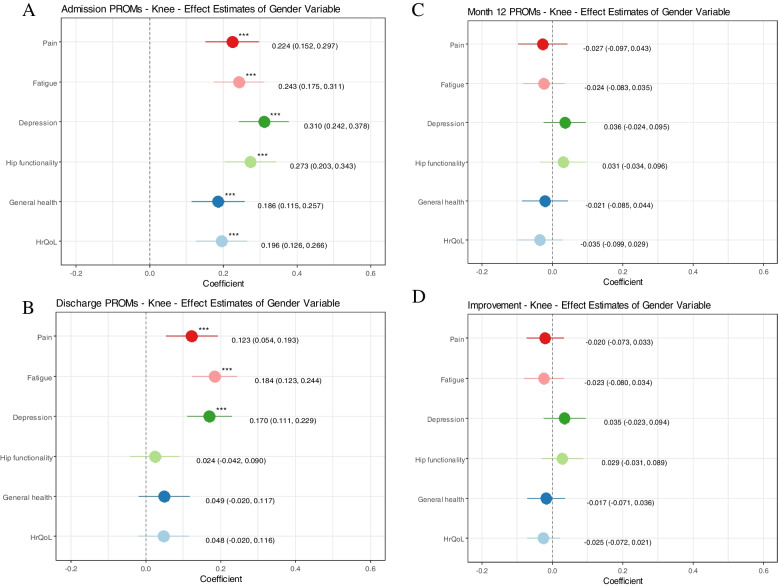
Knee arthroplasty: Explanatory power of the patients’ gender on the z-score standardized Patient-reported Outcomes (PROs) per measurement time. All graphs: the outcome variables (PROs) are transformed into z-scores and adjusted in their directionality so that all scores indicate better health in men if the coefficient is positive; HrQoL = Health-related quality of life. **Graph A** - admission: results of multivariate linear regression models controlling for age, education, BMI, living situation and nine comorbidities. **Graph B** - discharge: results of multivariate linear regression models controlling for age, Body Mass Index (BMI), the respective PRO at admission, the hospital, the mobilization after surgery, the experience of the main surgeon and nine comorbidities. **Graph C** - month 12: results of multivariate linear regression models controlling for age, BMI, the respective PRO at admission, the mobilization after surgery and the experience of the main surgeon, the monitoring group, the rehabilitation form and nine comorbidities. **Graph D** - PRO-improvement: results of multivariate linear regression models controlling for age, BMI, the respective PRO at admission, the mobilization after surgery and the experience of the main surgeon, the monitoring group, the rehabilitation form and nine comorbidities

The gender health gap, after adjusting for confounders through the multivariate linear regression models is illustrated in Figs. 2 and 3. The results are visualized using z-scores, depicting standard deviations from the mean, to make the PROs visually comparable on one scale. Positive effect estimates indicate better health in men. Significantly better health in men can be observed in all health dimensions pre-HA (KA) with standard deviations from the mean of 0.252 (0.224) for pain, 0.353 (0.243) for fatigue, 0.327 (0.310) for depression, 0.336 (0.273) for functionality, 0.177 (0.186) for general health and 0.266 (0.196) for HrQoL (Figs. 2A and 3A). Better health in men was observable at hospital discharge after HA (KA) for fatigue 0.162 (0.184) and depression 0.129 (0.170) and after HA by 0.203 in hip functionality and in pain by 0.123 after KA (Figs. 2B and 3B). The other health dimensions do not show significant differences at the *p* < 0.05 value level. 12-months post-surgery the only significant difference can be observed in men undergoing HA experiencing slightly worse fatigue than women by -0.081 (Figs. 2C and 3C). Similarly, the only difference in the PRO-improvement that is significant but not clinically meaningful is the difference in fatigue (-0.075) (Figs. 2D and 3D).

## Discussion

In this retrospective cohort study of HA and KA patients, we found that observed differences between genders were present but rather small in all health domains and differences reduced over time. Women reported significantly worse PROs at hospital admission in all examined health domains in both HA and KA patients when adjusting for patient and treatment characteristics. Whereas the raw unadjusted health gap shows that the point difference between genders reduces over time, while remaining significantly different at all observed times (besides pain 12-months post HA), the adjusted analyses showed that a significant gender health gap disappeared for some health domains at discharge (general health and HrQoL in both, HA and KA, physical functioning in KA and pain in HA) and all health domains 12-months post-surgery besides fatigue in HA patients (where the effect direction reverses). The gender differences in PRO-improvements can be considered not meaningful based on specified MCIDs. Unfortunately, no cutoff points exist that specify which between-group differences per measurement time (admission, discharge, month 12) can be considered meaningful, demanding future research.

The main analyses (multivariate regressions) of health differences at month 12, showcases the gender difference in individual health gain, meaning that a woman undergoing HA/KA has the same individual health gain (in all PROs except fatigue in HA patients) as a man if baseline PROs were the same (other patient and treatment variables controlled for). Putting this into perspective of the raw analyses reflecting women’s worse average admission and similar 12-month health status relative to men means on average women undergoing HA/KA in this study population have higher health gains than men. Women, e.g., experience around double the fatigue symptom improvement than men. Hence, the relationship between admission PROs and month 12 PROs in the context of HA/KA is similar for both genders but the observed distribution of health states per gender per timepoint is not.

The fact that men experience better overall, mental, and physical health prior to surgery while reaching similar self-reported health status 12-months post-surgery might be an indicator of men receiving surgery earlier than necessary or women receiving surgery later than necessary. It could also indicate that women and men are systematically different in reaching the right time for surgery at different health impairment levels. While there are studies showing gender health differences in the general population (e.g.Bloom et al. [35]), we assume that the health gap observed in the HA/KA population goes beyond this difference as the gender health gap in the HA/KA population reduces over time after surgery. Whether the observed differences are based on individual patient decisions, guidance from care providers or other factors is unclear and requires further research and care decision-making sensitive to gender differences. Hypotheses are presented in some papers and include women being referred to a surgeon only with a higher degree of disability [3, 36], women undergoing surgery with a higher age [37], and the unwillingness to accept surgery from the patient’s side [38–40]. As summarized by Novicoff and Saleh, women express more concerns regarding the risks of treatment and the disruption of their family-role [41]. In the context of the German healthcare system, evaluations from the national registry showed that there are higher risks of endoprosthesis failure due to a higher infection risk in men, which could explain these findings as well e.g. if women at risk for infection avoid surgery or men improve less on average due to higher infection risks [42]. Moreover, research on gender stereotypes also pointed to reporting and interpretation differences in pain perception, with women’s pain being judged as less severe by physicians than men’s pain when the descriptions of pain were the same [43]. In addition, Samulowitz et al. [44] and Mogil et al. [45] pointed out that women and men are brought up to express pain differently which can change their biological response to pain and their willingness to report it. However, as Moretti et al. [46] noted, gender is just one aspect of the multifactorial influences on outcomes and hence needs to be viewed from an intersectional lens when addressing the gender health gap. Potential reasons for gender differences stated by Tannenbaum et al. [47] that can be excluded based on the design of our study are firstly the experimenter-participant interaction as PROs are self-reported and secondly product-participant interaction as all questionnaires were validated in men and women. As the EQ-5D-5L is the only preference-based PROM in our study, the preference weights from the German value set might have however led to overemphasizing health dimensions that men are doing better in for the HrQoL assessment as suggested by Bischof et al. [48].

Based on the findings of this study, several implications for clinical practice can be drawn. Patients identifying as women might need more specific attention in the pre- and intraoperative phases to improve health outcomes as argued by Solarino et al. [49]. Both genders might also need a closer evaluation at which health and potentially arthritis status a HA or KA is beneficial to reduce low-value care. The implementation and evaluation of PROs before surgery could be one way to assess the optimal moment to perform surgery as shown by Tew et al. [50]. In light of described biases in physician–patient interaction, pre-surgery PROs have the benefit of objectifying the health assessment. Studies often include PROs in the form of the change in PRO from before to after therapy. As shown in this paper, looking only at the health change over time, and not the baseline health status, can lead to clouded interpretations. In the case of HA and KA, the results would indicate that women benefit more from surgery, however this is driven mainly by pre-surgery PRO levels. Looking at PROs before an intervention can point out improvement potential in the treatment of subgroups, e.g., more suggestions for alternative treatment options for men if their PROs are above a certain health threshold.

This study has several strengths and limitations. It specifies the gender health gap per observation time across different health domains for many PROs while controlling for known confounders. It moreover covers a large study population across different hospitals in Germany. Since there is no way to randomize for gender, controlling for confounders is the only way to digest the health difference due to gender. However, this study cannot control for some confounders or identify other reasons, e.g., for the pre-surgery health gap, like treatments pre-hospital admission, expectations from patients, or previous recommendation for surgery or arthritis progression in the affected joint. Similarly, there was no information available on the follow-up appointments besides the rehabilitation and PRO-monitoring, which could also explain the disappearing health gap at 12-months (e.g. if men are improving less because they miss follow-up care). We cannot say for certain whether men or women are closer to the optimum of receiving surgery when indicated. In addition, the gender variable was only assessed via three different answer options in the digital platform “man”, “woman”, “other”, whereas more and clearer gender options should have been represented in the study.

## Conclusion

This study shows that there are small differences across health domains between men and women undergoing HA/KA that reduce over time. We observed a significant gender health gap at admission to the hospital pre-HA and KA with men reporting better health status for pain, fatigue, depression, functionality, general health and HrQoL when controlling for various patient characteristics and comorbidities. At discharge from the hospital, the gender health gap reduced, and showed significant differences only for depression and fatigue in HA and KA, pain in KA and functionality in HA patients. 12-months post-surgery, the gap disappeared and even reversed in fatigue in HA patients, where men showed significantly worse scores. The difference in PRO-improvement were small and not clinically meaningful. To understand whether the differences, per measurement time, are meaningful to patients, further research is needed to identify cut-off points that go beyond existing ones only applicable to PRO-improvements. Men experiencing better health at hospital admission while reaching similar health levels 12-months post-surgery, when accounting for the pre-surgery health differences, might be an indicator of men receiving surgery earlier than necessary or women receiving surgery later than necessary, or both. Many studies only present the improvement in PROs over time, thereby missing pre-intervention gender differences, which could be an important target for improvement initiatives in patient-centric care.

### Supplementary Information

**Additional file 1:** **Table S1.** Descriptive statistics of the study population for additional variables (n = 6,803). **Table S2.** Summary statistics for PROs pre-surgery, at discharge and 12-months post-surgery and as change for hip arthroplasty patients per gender. **Table S3.** Summary statistics for PROs pre-surgery, at discharge and 12-months post-surgery and as change for knee arthroplasty patients per gender.

## Data Availability

The datasets generated and/or analysed during the current study are not publicly available due to the German data protection law but are available from the corresponding author on reasonable request.

## Abbreviations

BMI: Body Mass Index
EQ-VAS: European Quality of Life Visual Analogue Scale
EQ-5D-5L: 5-level EQ-5D version
HA: Hip Arthroplasty
HOOS-PS: Hip Disability and Osteoarthritis Outcome Score-Physical Function Short form
HrQoL: Health-related Quality of Life
KA: Knee Arthroplasty
KOOS-PS: Knee Injury and Osteoarthritis Outcome Score-Physical Function Short form
PROMIS-D-SF: Patient-Reported Outcomes Measurement Information System Depression Short Form
PROMIS-F-SF: Patient Reported Outcome Measurement Information System Fatigue-Short Form
PROM(s): Patient Reported Outcome Measure(s)
PRO(s): Patient Reported Outcome(s)
