# Supplementary material for: Gender health gap pre- and post-joint arthroplasty: identifying affected patient-reported health domains
Source: Int J Equity Health. 2024 Feb 27;23:44. doi: 10.1186/s12939-024-02131-5 (PMC10900674; doi:10.1186/s12939-024-02131-5)
Supplement: Supplementary file 1 — Additional file 1: Table S1. Descriptive statistics of the study population for additional variables (n = 6,803). Table S2. Summary statistics for PROs pre-surgery, at discharge and 12-months post-surgery and as change for hip arthroplasty patients per gender. Table S3. Summary statistics for PROs pre-surgery, at discharge and 12-months post-surgery and as change for knee arthroplasty patients per gender. [file 12939_2024_2131_MOESM1_ESM.docx]

**Supplementary file**

***Table S1:*** *Descriptive statistics of the study population for additional variables (n = 6,803)*

|  | Hip replacement patients | | p^b^ | Knee replacement patients | | p^b^ |
| --- | --- | --- | --- | --- | --- | --- |
|  | men (N=1,627) | women (N=2,066) |  | men  (N=1,430) | women  (N=1,680) |  |
| Comorbidities | | | | | | |

| Heart-related (yes) | 332  (20.41%) | 233  (11.28%) | < 0.001 *** | 252  (17.62%) | 204  (12.14%) | < 0.001  *** |
| --- | --- | --- | --- | --- | --- | --- |
| Blood-related (yes) | 916  (56.30%) | 1043  (50.48%) | < 0.001 *** | 935  (65.38%) | 1092  (65.00%) | 0.823 |
| Stroke  (yes) | 0  (0.00%) | 0  (0.00%) | / | 0  (0.00%) | 0  (0.00%) | / |
| Circulation-related (yes) | 0  (0.00%) | 0  (0.00%) | / | 0  (0.00%) | 0  (0.00%) | / |
| Lung-related (yes) | 145  (8.91%) | 237  (11.47%) | 0.011  ** | 144  (10.07%) | 249  (14.82%) | < 0.001  *** |
| Diabetes  (yes) | 192  (11.80%) | 160  (7.74%) | < 0.001  *** | 160  (11.19%) | 158  (9.40%) | 0.102 |
| Neurological (yes) | 41  (2.52%) | 68  (3.29%) | 0.1691 | 42  (2.94%) | 74  (4.40%) | 0.031  ** |
| Cancer  (yes) | 157  (9.65%) | 137  (6.63%) | < 0.001 *** | 88  (6.15%) | 98  (5.83%) | 0.707 |
| Depression (yes) | 99  (6.08%) | 224  (10.84%) | < 0.001 *** | 71  (4.97%) | 226  (13.45%) | < 0.001  *** |
| Back-related (yes) | 448  (27.54%) | 821  (39.74%) | < 0.001 *** | 393  (27.48%) | 687  (40.89%) | < 0.001  *** |
| Arthritis  (yes) | 130  (7.99%) | 260  (12.58%) | < 0.001 *** | 146  (10.21%) | 251  (14.94%) | < 0.001  *** |
| *PRO^a^-remote monitoring group* | | | | | | |
| *monitoring* | 821  (50.46%) | 1,029 (49.81%) | 0.692 | 720  (50.35%) | 844  (50.24%) | 0.950 |
| *no monitoring* | 806  (49.54%) | 1,037 (50.19%) |  | 710  (49.65%) | 836  (49.76%) |  |
| Mobilization after surgery | | | | | | |

| within 6 hours | 775  (47.63%) | 931 (45.06%) | 0.076 | 659  (46.08%) | 766  (45.60%) | 0.755 |
| --- | --- | --- | --- | --- | --- | --- |
| within 12 hours | 453  (27.84%) | 600 (29.04%) |  | 404  (28.25%) | 481  (28.63%) |  |
| within 24 hours | 367  (22.56%) | 480 (23.23%) |  | 320  (22.38%) | 367  (21.85%) |  |
| within 48 hours | 27  (1.66%) | 34  (1.65%) |  | 39  (2.73%) | 50  (2.98%) |  |
| after 48 hours | 5  (0.31%) | 21  (1.02%) |  | 8  (0.56%) | 16  (0.95%) |  |

*^a^PRO = patient-reported outcome*

*^b^significance levels were set at p<0.1*, p<0.05** and p<0.01****

***Table S2:*** *Summary statistics for PROs pre-surgery, at discharge and 12-months post-surgery and as change for hip arthroplasty patients per gender*

| gender | men  (n = 1,627) | | | women  (n =2,066) | | | women vs. men difference of means  (gender health gap) | |
| --- | --- | --- | --- | --- | --- | --- | --- | --- |
| variable^a^ | Mean | SD | Median | Mean | SD | Median | points | p-value^b^ |
| admission | | | | | | | | |
| EQ-5D-5L | 0.644 | 0.239 | 0.744 | 0.562 | 0.269 | 0.634 | -0.081 | < 0.001 |
| EQ-VAS | 59.14 | 19.90 | 60.00 | 54.96 | 19.67 | 54.00 | -4.18 | < 0.001 |
| HOOS-PS | 44.29 | 15.50 | 41.70 | 50.43 | 16 | 50.80 | 6.13 | < 0.001 |
| PROMIS-D-SF | 47.96 | 7.78 | 49.00 | 51.20 | 8.4 | 51.80 | 3.25 | < 0.001 |
| PROMIS-F-SF | 46.92 | 9.20 | 49.00 | 50.97 | 10 | 51.00 | 4.05 | < 0.001 |
| Pain in joint | 6.09 | 2.19 | 7.00 | 6.72 | 2.05 | 7.00 | 0.63 | < 0.001 |
| discharge | | | | | | | | |
| EQ-5D-5L | 0.780 | 0.170 | 0.816 | 0.755 | 0.189 | 0.800 | -0.025 | < 0.001 |
| EQ-VAS | 65.87 | 18.08 | 70.00 | 63.69 | 18.10 | 66.00 | -2.18 | < 0.001 |
| HOOS-PS | 43.02 | 16.49 | 41.70 | 48.90 | 17.77 | 50.80 | 5.88 | < 0.001 |
| PROMIS-D-SF | 47.13 | 7.15 | 41.00 | 49.63 | 7.79 | 49.00 | 2.50 | < 0.001 |
| PROMIS-F-SF | 46.92 | 8.53 | 48.60 | 50.07 | 9.36 | 48.60 | 3.15 | < 0.001 |
| Pain in joint | 3.35 | 2.08 | 3.00 | 3.62 | 2.18 | 3.50 | 0.27 | < 0.001 |
| month 12 | | | | | | | | |
| EQ-5D-5L | 0.897 | 0.148 | 0.943 | 0.876 | 0.171 | 0.918 | -0.021 | < 0.001 |
| EQ-VAS | 76.11 | 17.40 | 80.00 | 74.84 | 17.80 | 80.00 | -1.27 | 0.009 |
| HOOS-PS | 13.10 | 12.93 | 8. 80 | 14.99 | 14.05 | 12.57 | 1.89 | < 0.001 |
| PROMIS-D-SF | 46.10 | 6.93 | 41.00 | 47.79 | 7.60 | 45.44 | 1.70 | < 0.001 |
| PROMIS-F-SF | 44.25 | 8.13 | 46.00 | 45.51 | 8.84 | 46.00 | 1.26 | < 0.001 |
| Pain in joint | 0.91 | 1.60 | 0.00 | 0.95 | 1.69 | 0.00 | 0.04 | 0.585 |
| improvement (change from admission to month 12) | | | | | | | | |
| EQ-5D-5L | 0.252 | 0.246 | 0.187 | 0.313 | 0.275 | 0.250 | 0.061 | < 0.001 NCM^c^ |
| EQ-VAS | 16.97 | 22.54 | 15.00 | 19.88 | 22.31 | 20.00 | 2.91 | < 0.001 NCM^c^ |
| HOOS-PS | -31.19 | 17.09 | -30.40 | -35.44 | 18.22 | -35.90 | -4.24 | < 0.001 NCM^c^ |
| PROMIS-D-SF | -1.86 | 7.44 | 0.00 | -3.41 | 8.19 | -1.70 | -1.55 | < 0.001 NCM^c^ |
| PROMIS-F-SF | -2.68 | 8.60 | -2.40 | -5.46 | 9.63 | -5.16 | -2.79 | < 0.001  CM^c^ |
| Pain in joint | -5.19 | 2.57 | -5.00 | -5.78 | 2.52 | -6.00 | -0.59 | < 0.001  NCM^c^ |

*^a^ The score ranges for the PROMs are: -0.661 to 1.0 for the EQ-5D-5L, 0-100 for the EQ-VAS, HOOS-PS and KOOS-PS, 33.7-75.8 for the PROMIS-F-SF, 41-79.4 for the PROMIS-D-SF and 0-10 for pain. The EQ-5D-5L is reported with three digits after the decimal point due to the small score range ^b^ Calculated based on the Mann-Whitney U test for the admission, discharge and month 12 values and with a two-sided t-test for the change from admission to month 12.*

^c^ *CM indicates clinically meaningful, based on minimally clinically important difference (MCID) thresholds for PRO-improvement, NCM indicates not clinically meaningful*

***Table S3:*** *Summary statistics for PROs pre-surgery, at discharge and 12-months post-surgery and as change for knee arthroplasty patients per gender*

| gender | men  (n = 1,430) | | | women  (n = 1,680) | | | women vs. men difference of means  (gender health gap) | |
| --- | --- | --- | --- | --- | --- | --- | --- | --- |
| variable^a^ | Mean | SD | Median | Mean | SD | Median | points | p-value^b^ |
| admission | | | | | | | | |
| EQ-5D-5L | 0.665 | 0.230 | 0.752 | 0.589 | 0.262 | 0.666 | -0.076 | < 0.001 |
| EQ-VAS | 61.09 | 19.29 | 62.00 | 55.91 | 18.92 | 53.50 | -5.19 | < 0.001 |
| KOOS-PS | 40.62 | 12.67 | 40.30 | 45.13 | 12.35 | 44.00 | 4.51 | < 0.001 |
| PROMIS-D-SF | 47.42 | 7.69 | 41.00 | 51.04 | 8.39 | 51.80 | 3.61 | < 0.001 |
| PROMIS-F-SF | 46.28 | 9.51 | 46.00 | 49.98 | 9.80 | 48.60 | 3.61 | < 0.001 |
| Pain in joint | 6.49 | 2.04 | 7.00 | 7.04 | 1.93 | 7.00 | 0.55 | < 0.001 |
| discharge | | | | | | | | |
| EQ-5D-5L | 0.754 | 0.192 | 0.805 | 0.724 | 0.214 | 0.788 | -0.030 | < 0.001 |
| EQ-VAS | 63.34 | 17.97 | 66.00 | 60.54 | 17.56 | 60.00 | -2.80 | < 0.001 |
| KOOS-PS | 43.46 | 11.52 | 42.00 | 45.67 | 12.07 | 44.00 | 2.21 | < 0.001 |
| PROMIS-D-SF | 46.81 | 7.20 | 41.00 | 49.98 | 8.12 | 51.80 | 3.17 | < 0.001 |
| PROMIS-F-SF | 47.11 | 8.84 | 48.60 | 50.50 | 8.989 | 48.60 | 3.39 | < 0.001 |
| Pain in joint | 4.34 | 2.18 | 4.00 | 4.80 | 2.26 | 5.00 | 0.46 | < 0.001 |
| month 12 | | | | | | | | |
| EQ-5D-5L | 0.869 | 0.172 | 0.917 | 0.838 | 0.197 | 0.901 | -0.030 | < 0.001 |
| EQ-VAS | 73.30 | 17.29 | 79.00 | 70.47 | 18.31 | 75.00 | -2.83 | < 0.001 |
| KOOS-PS | 24.13 | 13.62 | 22.05 | 27.09 | 12.49 | 27.50 | 2.96 | < 0.001 |
| PROMIS-D-SF | 46.03 | 7.05 | 41.00 | 48.54 | 7.99 | 47.81 | 2.51 | < 0.001 |
| PROMIS-F-SF | 44.62 | 8.59 | 46.00 | 46.63 | 8.86 | 46.20 | 2.00 | < 0.001 |
| Pain in joint | 1.65 | 1.89 | 1.00 | 1.83 | 2.01 | 1.00 | 0.18 | 0.023 |
| improvement (change from admission to month 12) | | | | | | | | |
| EQ-5D-5L | 0.204 | 0.240 | 0.146 | 0.250 | 0.266 | 0.187 | 0.046 | < 0.001  NCM^c^ |
| EQ-VAS | 12.21 | 20.86 | 10.00 | 14.56 | 22.16 | 14.00 | 2.36 | 0.002  NCM^c^ |
| KOOS-PS | -16.49 | 14.22 | -16.40 | -18.04 | 13.99 | -17.10 | -1.55 | 0.002  NCM^c^ |
| PROMIS-D-SF | -1.39 | 7.39 | 0.00 | -2.497 | 8.012 | 0.00 | -1.11 | < 0.001  NCM^c^ |
| PROMIS-F-SF | -1.66 | 8.86 | 0.00 | -3.266 | 9.21 | -2.60 | -1.61 | < 0.001 NCM^c^ |
| Pain in joint | -4.84 | 2.50 | -5.00 | -5.21 | 2.63 | -5.00 | -0.36 | < 0.001  NCM^c^ |

*^a^ The score ranges for the PROMs are: -0.661 to 1.0 for the EQ-5D-5L, 0-100 for the EQ-VAS, HOOS-PS and KOOS-PS, 33.7-75.8 for the PROMIS-F-SF, 41-79.4 for the PROMIS-D-SF and 0-10 for pain. The EQ-5D-5L is reported with three digits after the decimal point due to the small score range*

*^b^ Calculated based on the Mann-Whitney U test and with a two-sided t-test for the change from admission to month 12.*

^c^ *CM indicates clinically meaningful, based on minimally clinically important difference (MCID) thresholds for PRO-improvement, NCM indicates not clinically meaningful*
